# Enhanced cross-recognition of SARS-CoV-2 Omicron variant by peptide vaccine-induced antibodies

**DOI:** 10.3389/fimmu.2022.1044025

**Published:** 2023-01-24

**Authors:** Belén Aparicio, Marta Ruiz, Noelia Casares, Leyre Silva, Josune Egea, Patricia Pérez, Guillermo Albericio, Mariano Esteban, Juan García-Arriaza, Juan J. Lasarte, Pablo Sarobe

**Affiliations:** ^1^ Centro de Investigación Médica Aplicada (CIMA), Universidad de Navarra, Pamplona, Spain; ^2^ Centro de Investigación Biomédica en Red de Enfermedades Hepáticas y Digestivas (CIBEREHD), Pamplona, Spain; ^3^ Instituto de Investigaciones Sanitarias de Navarra (IdiSNA), Pamplona, Spain; ^4^ Centro Nacional de Biotecnología (CNB), Consejo Superior de Investigaciones Científicas (CSIC), Madrid, Spain; ^5^ Centro de Investigación Biomédica en Red de Enfermedades Infecciosas (CIBERINFEC), Madrid, Spain

**Keywords:** SARS-CoV-2, peptide vaccine, Omicron variant, cross-recognizing antibodies, conserved regions

## Abstract

Current vaccines against SARS-CoV-2, based on the original Wuhan sequence, induce antibodies with different degrees of cross-recognition of new viral variants of concern. Despite potent responses generated in vaccinated and infected individuals, the Omicron (B.1.1.529) variant causes breakthrough infections, facilitating viral transmission. We previously reported a vaccine based on a cyclic peptide containing the 446-488 S1 sequence (446-488cc) of the SARS-CoV-2 spike (S) protein from Wuhan isolate. To provide the best immunity against Omicron, here we compared Omicron-specific immunity induced by a Wuhan-based 446-488cc peptide, by a Wuhan-based recombinant receptor-binding domain (RBD) vaccine and by a new 446-488cc peptide vaccine based on the Omicron sequence. Antibodies induced by Wuhan peptide 446-488cc in three murine strains not only recognized the Wuhan and Omicron 446-488 peptides similarly, but also Wuhan and Omicron RBD protein variants. By contrast, antibodies induced by the Wuhan recombinant RBD vaccine showed a much poorer cross-reactivity for the Omicron RBD despite similar recognition of Wuhan and Omicron peptide variants. Finally, although the Omicron-based 446-488cc peptide vaccine was poorly immunogenic in mice due to the loss of T cell epitopes, co-immunization with Omicron peptide 446-488cc and exogenous T cell epitopes induced strong cross-reactive antibodies that neutralized Omicron SARS-CoV-2 virus. Since mutations occurring within this sequence do not alter T cell epitopes in humans, these results indicate the robust immunogenicity of 446-488cc-based peptide vaccines that induce antibodies with a high cross-recognition capacity against Omicron, and suggest that this sequence could be included in future vaccines targeting the Omicron variant.

## 1 Introduction

The emergence of new SARS-CoV-2 variants of concern may affect the protective efficacy of vaccines. Current vaccines, based on the original Wuhan sequence, induce antibodies with a poorer recognition and neutralization capacity against the Omicron (B.1.1.529) variant (1–3). However, T cell responses induced by vaccination or viral infection cross-recognize this variant (4, 5). Although fully vaccinated individuals present no or milder symptoms and lower hospitalization risk when exposed to this variant, breakthrough infections cannot be avoided (6–8), with the risk of viral transmission. Moreover, infections caused by Omicron provide a poorer antibody boost than those induced by other variants (9). Therefore, despite partial protection achieved by current vaccines or after infection, vaccines able to induce antibodies with wider coverage are urgently needed. We recently identified the amino acid region 446-488 in the receptor-binding domain (RBD) of SARS-CoV-2 S1 as a sequence containing T and B cell epitopes that, when used as a cyclized peptide vaccine (446-488cc), elicited cellular and humoral neutralizing responses that protected mice against SARS-CoV-2 infection (10). Moreover, this vaccine induced antibodies that recognized other viral variants. Omicron variant harbors several mutations, including more than 20 point mutations and deletions in S1 protein (11), the target antigen in current vaccines. Some of these mutations (G446S, L452R, S477N, T478K, E484A) lie within the 446-488 region, potentially modifying the capacity of antibodies to recognize new variants. Omicron-based vaccines have been proposed to induce more specific responses against the currently existing virus. Thus, in order to obtain information about the usefulness of 446-488cc-based vaccines against the Omicron variant we analyzed immunity induced by the original Wuhan-based peptide vaccine or by a new Omicron-based peptide vaccine, measuring antibody responses against Omicron.

## 2 Methods

### 2.1 Antigens

**Figure 1:**
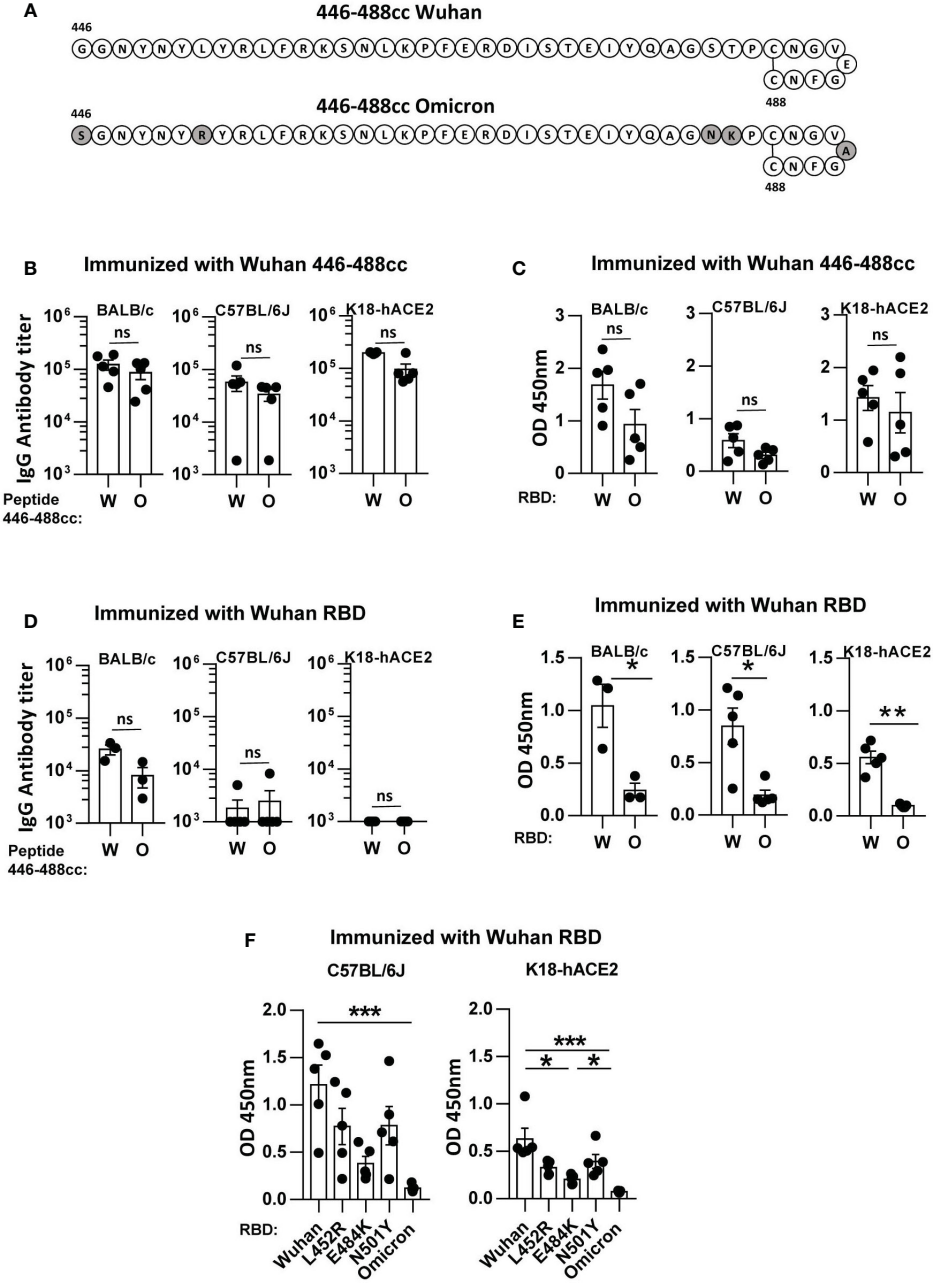
Cross-recognition of SARS-CoV-2 Wuhan and Omicron antigens by binding IgG antibodies induced by a Wuhan 446-488c peptide vaccine and by a Wuhan recombinant RBD vaccine in immunized mice. **(A)** Schematic diagram of peptides 446-488cc belonging to Wuhan and Omicron variants, with indication of the amino acid sequence. Amino acid mutations in the Omicron peptide, compared to the Wuhan sequence, are highlighted in grey. BALB/c, C57BL/6J and K18-hACE2 mice were immunized with the Wuhan 446-488cc peptide vaccine (n=5/group) **(B, C)** or with a Wuhan RBD protein vaccine (n=3-5/group) **(D, E)**. Sera obtained after boosting with the same vaccine were used to test by ELISA recognition of Wuhan and Omicron 446-488cc peptides **(B, D)** or Wuhan and Omicron RBDs **(C, E)** by binding IgG antibodies. **(F)** Recognition of different RBD variants by sera obtained from mice immunized with Wuhan RBD. Bars show mean +/- SEM ns, not significant; *, P<0.05; **, P<0.01; ***, P<0.001. W, Wuhan; O, Omicron.

Peptides 446-488cc (containing a disulfide bridge between cysteines 480 and 488) corresponding to the original Wuhan sequence and Omicron variant (containing the common mutations S477N, T478K and E484A, plus mutations G446S and L452R, found in variants BA.1 and BA.4/5, respectively) (12, 13) (purity >90%) (**Figure 1A**), as well as 15-mer peptides containing T cell epitopes were purchased from Genecust (Boynes, France). RBD Wuhan (Cat. No. Z03483-100) and Omicron (variant BA.1; Cat. No. Z03730-100) proteins, as well as those RBDs with mutations E484K (Cat. No. Z03535-100), N501Y (Cat. No. Z03533-100) or L452R (Cat. No. Z03603), all expressed in human cells, were purchased from GenScript (Leiden, The Netherlands).

### 2.2 Mice

BALB/c and C57BL/6J mice (8 weeks old) were obtained from Envigo (Barcelona, Spain). K18-hACE2 mice (B6.Cg-Tg(K18-ACE2)2Prlmn/J) (8 weeks old) expressing human angiotensin converting enzyme 2 (ACE-2) were purchased from The Jackson Laboratory (Bar Harbor, ME). Mice were maintained in pathogen-free conditions according to the guidelines for animal care of our Institutional Review Board (protocol 025-20). Animal procedures conformed with international guidelines and with Spanish law under the Royal Decree (RD 53/2013).

### 2.3 Immunization

For peptide immunization mice received by intramuscular route 110 μg of peptide 446-488cc (Wuhan or Omicron sequences) in combination with 10 μg of the CpG-containing oligodeoxynucleotide ODN1018 (Sigma; Darmstadt, Germany) and 50 μg of alum (*In vivo*gen; Toulouse, France) and they were boosted similarly at day 21. In other cases, mice received intraperitoneally the same dose of the Omicron-based peptide in combination with 50 μg of T helper peptides FIS (FISEAIIHVLHSR) for BALB/c mice and PADRE (AKFVAAWTLKAAA) for C57BL/6J mice, emulsified in complete Freund’s adjuvant (CFA) (Difco Laboratories; Detroit, MI). They were boosted at days 30 and 45 with the same dose of antigens in incomplete Freund’s adjuvant (IFA). For RBD immunization mice received intraperitoneally 1 μg of RBD protein emulsified in CFA and were boosted with antigens in IFA as above.

### 2.4 ELISA

Antigen recognition by sera from vaccinated mice was carried out by Enzyme-Linked ImmunoSorbent Assay (ELISA) as described (14). Briefly, plates were coated with peptides 446-488cc containing the Wuhan or Omicron sequences (1 µg/well) or RBD proteins (original Wuhan RBD, Omicron variant RBD and RBD variants with single mutations L452R, E484K or N501Y; in all cases 0.1 µg/well) and after washing and blockade, different serial serum dilutions were added and incubated for 2 h at room temperature. After several washes, IgG antibodies were detected by incubation with a biotinylated goat anti-murine IgG antibody and streptavidin peroxidase. Serum endpoint titers were calculated for each mouse as the last dilution with an optical density at 450 nm above that obtained with control naïve sera plus 2 standard deviations.

### 2.5 ELISPOT

T-cell responses induced by vaccines were measured by using an Interferon (IFN)-γ Enzyme-Linked ImmunoSpot (ELISPOT) assay (BD-Biosciences; San Diego, CA) as described (10). Briefly, spleens from immunized mice were individually homogenized and, after lysing erythrocytes with red blood cell (RBC) lysing buffer, splenocytes (8 x 10^5^/well) were stimulated for 24 h with vaccine peptides or previously defined T cell epitopes (10 μg/ml). In some assays, purified CD4 or CD8 T cells (1.6 x 10^5^ or 0.8 x 10^5^ cells/well, according to their proportions in total spleen cells) obtained using negative selection kits (Cat. No. 130-104-454 and 130-104-075, respectively; Miltenyi Biotec, Germany) were co-cultured with irradiated (10000 rads) splenocytes obtained from naïve unimmunized mice to reach a total 8 x 10^5^ cells/well. Control wells without peptide stimulation were included in all assays. After washing and incubation with detection antibody for 2 h, spots were developed by using 3-Amino-9-ethylcarbazole substrate. Spot-forming cells were counted with an ImmunoSpot automated counter (CTL-Immunospot; Bonn, Germany). Results were expressed as the difference between peptide-stimulated minus control wells.

### 2.6 Prediction of peptide binding to MHC molecules

Sequences corresponding to Wuhan or Omicron 446-488 peptides were uploaded in NetMHCpan 4.1 (https://services.healthtech.dtu.dk/service.php?NetMHCpan-4.1) and NetMHCIIpan 4.0 (https://services.healthtech.dtu.dk/service.php?NetMHCIIpan-4.0) to identify peptides with potential major histocompatibility complex (MHC) class I and class II binding capacity, respectively. For MHC class I binding, peptides with a length of 8 to 11 amino acids were considered, and were tested against a panel of 9 murine and 12 human alleles, selecting those with a % Rank < 0.5 as strong binders and those with a % Rank between 0.5 and 2 as weak binders. For MHC class II binding, 15-mer peptides were tested against 3 murine and 54 human alleles. Peptides with a % Rank < 2 were selected as strong binders, whereas a % Rank between 2 and 10 defined weak binders.

### 2.7 SARS-CoV-2 omicron neutralization experiments

Capacity of the sera obtained from BALB/c and C57BL/6J immunized mice to neutralize live SARS-CoV-2 B.1.1.529 Omicron BA.1 variant (hCoV-19/Belgium/rega-20174/2021, EPI_ISL_6794907; kindly provided by Dr. Robbert Boudewijns and Dr. Kai Dallmeier, KU Leuven, Belgium through an MTA agreement) (15) was measured using a microneutralization test (MNT) assay in a BSL-3 laboratory at the CNB-CSIC as described (16). Serially twofold diluted serum samples in DMEM-2% fetal bovine serum (FBS) medium were incubated at a 1:1 ratio with 200 tissue culture infectious dose 50 (TCID_50_) of SARS-CoV-2 Omicron BA.1 variant in 96-well tissue culture plates for 1 h at 37°C. Then, mixtures of serum samples and SARS-CoV-2 were added in triplicate to Vero-E6 TMPRSS2 cell monolayers seeded in 96-well plates at 2 x 10^4^ cells/well, and plates were incubated at 37°C, in a 5% CO_2_ incubator for 48 h. Then, cells were fixed with 10% formaldehyde for 1 h and stained with crystal violet. When plates were dried, crystal violet was diluted in H_2_O-1% sodium dodecyl sulfate (SDS) and optical density was measured in a luminometer at 570 nm. To obtain the neutralization titers, half maximal inhibitory concentration (IC_50_) and 95% confidence intervals (95% CI) were calculated using a nonlinear regression model fit with settings for log agonist versus normalized response curve using GraphPad Prism v9 Software.

### 2.8 Statistical analyses

They were performed with GraphPad Prism (GraphPad) v7. After checking for normality, T-tests or non-parametric tests were used. P<0.05 was taken to represent statistical significance.

## 3 Results

### 3.1 Wuhan peptide 446-488cc-induced IgG antibodies have a high cross-recognition of Omicron sequences

In a first set of experiments we analyzed the capacity of sera induced by immunization with Wuhan peptide 446-488cc to recognize antigens (peptide 446-488cc and RBD protein) belonging to Wuhan or Omicron variants. The Omicron 446-488cc peptide contained the common mutations S477N, T478K and E484A, plus mutations G446S and L452R, found in variants BA.1 and BA.4/5, respectively (12, 13) (**Figure 1A**). To this end we used sera from immunized BALB/c and C57BL/6J mice, as well as from transgenic K18-hACE2 mice, also vaccinated with this peptide in our previous viral protection experiments (10). In all murine strains, despite a slight decrease in binding IgG antibody titers against Omicron 446-488cc peptide (**Figure 1B**) and against Omicron RBD (**Figure 1C**), no statistically significant differences were observed when compared with the IgG antibody levels induced against Wuhan 446-488cc peptide or Wuhan RBD. These results are in agreement with our previous data regarding cross-recognition of peptides and RBD proteins from other variants after immunization with Wuhan peptide 446-488cc (10).

After analyzing peptide vaccine-induced IgG antibodies, we next compared these results with those obtained using a different set of sera, in this case those generated in mice immunized with a recombinant Wuhan RBD protein vaccine (amino acids 319-541 of S1). This would partially mimic the situation generated in vaccinated individuals. In this case, recognition of the Omicron 446-488cc peptide did not show statistically significant differences when compared with the Wuhan 446-488cc peptide in BALB/c mice. Interestingly, the Wuhan recombinant RBD vaccine barely induced IgG antibodies specifically recognizing Wuhan or Omicron 446-488cc peptide variants in C57BL/6J and K18-hACE2 mice (**Figure 1D**). However, when these Wuhan RBD-induced sera were tested against Wuhan and Omicron RBD variants, in all murine strains recognition of Omicron RBD was very poor and significantly lower than the IgG antibody levels against Wuhan RBD (**Figure 1E**).

We previously showed that Wuhan peptide 446-488cc-induced IgG antibodies could also recognize different RBD proteins with mutations L452R, E484K or N501Y (10). Thus, finally we tested the recognition of these RBD variants by Wuhan RBD-induced IgG antibodies from C57BL/6J and K18-hACE2 mice. We found that, in comparison to Wuhan RBD, although some differences were observed against these RBD variants (e.g. RBD E484K), Omicron RBD variant showed the poorest recognition (**Figure 1F**).

### 3.2 Immunogenicity of an Omicron sequence-based 446-488cc peptide vaccine in mice

**Figure 2:**
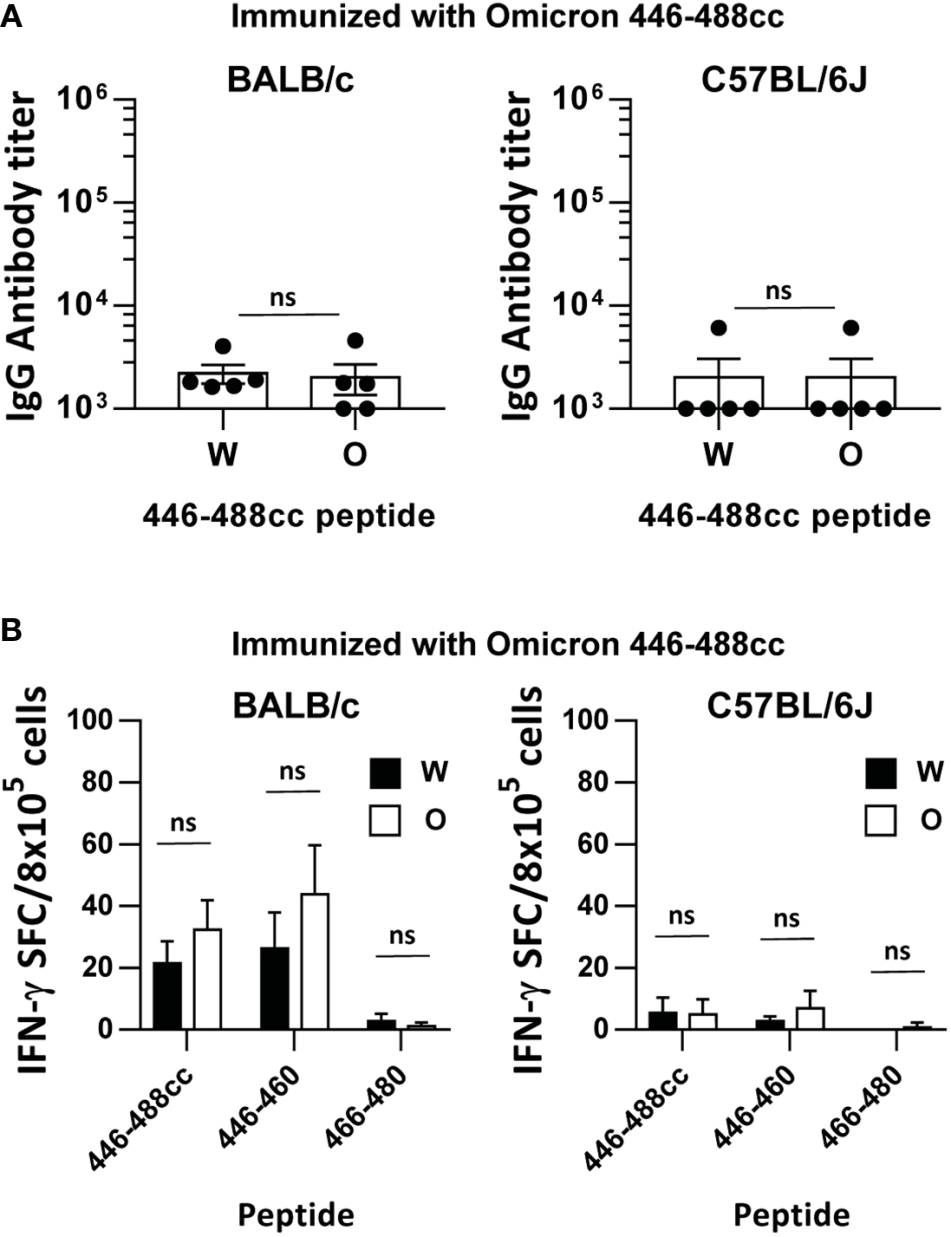
Immunogenicity of an Omicron sequence-based 446-488cc peptide vaccine in immunized mice. BALB/c and C57BL/6J mice were immunized with an Omicron-based 446-488cc peptide vaccine (n=5/group). **(A)** Binding IgG antibodies against Wuhan 446-488cc and Omicron 446-488cc peptides were measured by ELISA. **(B)** Splenocytes from these animals were stimulated *in vitro* with Wuhan and Omicron 446-488cc, 446-460 and 466-480 peptides, and T cell responses were determined by an IFN-γ ELISPOT assay. Results correspond to data after subtraction of values obtained in control wells without peptide. Bars show mean +/- SEM (ns, not significant). W, Wuhan; O, Omicron.

To elucidate if an Omicron sequence-based peptide vaccine would yield improved results against Omicron antigens, BALB/c and C57BL/6J mice were immunized with the Omicron peptide 446-488cc. Surprisingly, binding IgG antibody responses elicited by Omicron 446-488cc peptide against peptide 446-488cc from Wuhan or Omicron were much lower than those induced by the Wuhan 446-488cc peptide vaccine, with barely detectable responses in BALB/c mice, and only one animal with meaningful titers in C57BL/6J mice (**Figure 2A**). Analyses of T cells with capacity to recognize the whole 43-mer immunogen or previously identified 15-mer murine T cell epitopes, revealed that the Omicron peptide-based vaccine induced weak responses in BALB/c mice and almost no responses C57BL/6J mice, either against the Wuhan or Omicron-corresponding sequences (**Figure 2B**).

**Figure 3:**
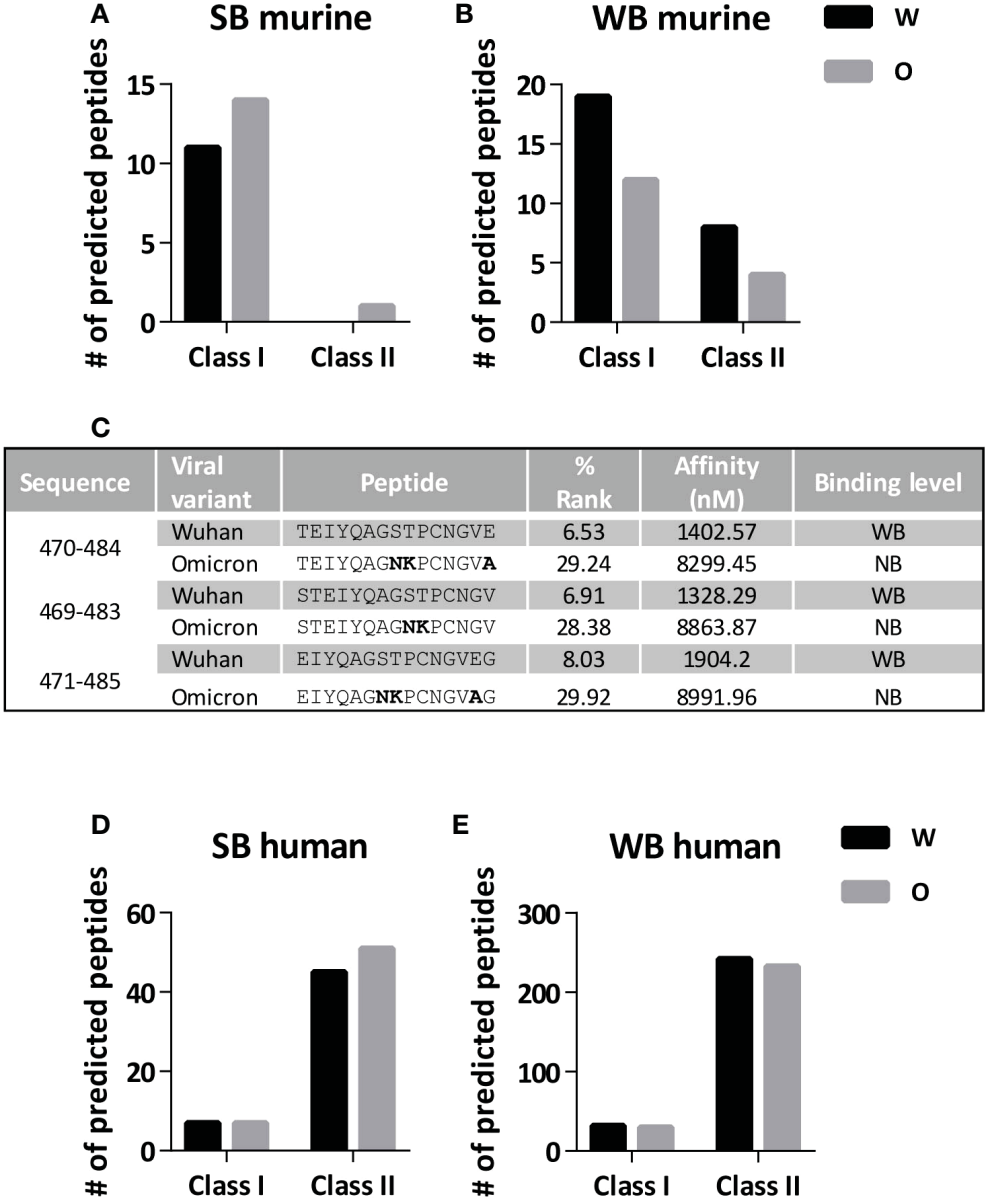
Prediction of MHC binder peptides in region 446-488 in Wuhan and Omicron variants. The number of strong binder (SB) and weak binder (WB) peptides contained in the 446-488 region of Wuhan and Omicron variants was determined by using MHC binding prediction algorithms. Number of SB peptides **(A)** and WB peptides **(B)** when considering a panel of murine MHC class I and II alleles. **(C)** Binding ability to murine I-A^b^ molecules (determined as % Rank and Affinity) of three peptides predicted as WB in the Wuhan sequence (NB; non-binder). Mutated residues are shown in bold. Number of SB peptides **(D)** and WB peptides **(E)** when considering a panel of human HLA class I and II alleles. W, Wuhan; O, Omicron.

To understand the lack of immunogenicity of the Omicron 446-488cc peptide vaccine, we analyzed the putative binding capacity of T cell epitopes contained within this region. By using prediction algorithms, we predicted binding to a panel of different murine MHC class I and II alleles. The number of the few strong binder (SB) peptides (for MHC class I and class II epitopes) remained almost unchanged when studying Wuhan and Omicron sequences (**Figure 3A**). However, when analyzing weak binder (WB) peptides, the Omicron variant contained nearly half of those present in the Wuhan sequence (**Figure 3B**), either for murine MHC class I or class II alleles. In this respect, whereas the number of binder peptides did not change when considering MHC molecules expressed by BALB/c, the three putative WB peptides of the Wuhan sequence presented by C57BL/6J MHC class II I-A^b^ molecules were lost in the case of the Omicron variant (**Figure 3C**), potentially explaining the lack of response in this murine strain. To elucidate whether these findings obtained in mice could be potentially translated into humans, equivalent binding predictions were carried out using a panel of human HLA class I and class II alleles. As opposed to murine MHC alleles, HLA binding prediction algorithms did not reveal differences between Wuhan and Omicron sequences, both in terms of SB and WB peptides, in HLA class I (**Figure 3D**) and II molecules (**Figure 3E**).

**Figure 4:**
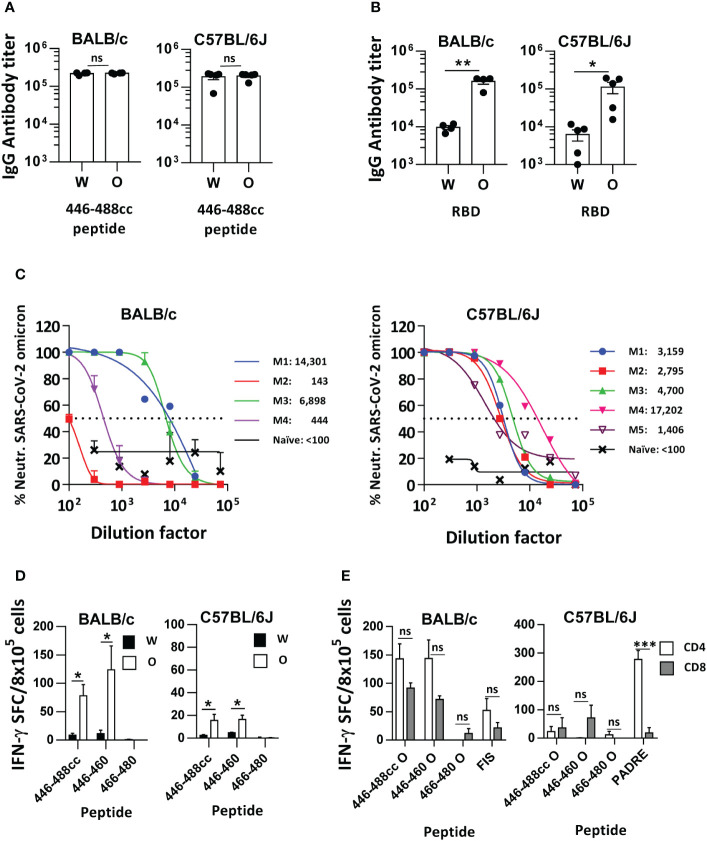
Immunogenicity of the Omicron sequence-based 446-488cc peptide vaccine co-administered with exogenous T helper epitopes in immunized mice. BALB/c and C57BL/6J mice were immunized with an Omicron-based 446-488cc peptide vaccine (n=4-5/group) plus exogenous T helper epitopes FIS or PADRE. Binding IgG antibodies against 446-488cc Wuhan and Omicron peptides **(A)** and against Wuhan and Omicron RBD proteins **(B)** were measured by ELISA. **(C)** Neutralizing activity of sera shown in A and B was tested against live Omicron SARS-CoV-2 by using serial dilutions. Results from individual mice (M1 to M4/5) with the corresponding 50% neutralization titer are also shown. Dotted line represents the 50% neutralization. As a control, pooled sera from 4-5 naïve BALB/c or C57BL/6J mice were also tested. **(D)** Splenocytes from these animals were stimulated *in vitro* with Wuhan and Omicron 446-488cc, 446-460 and 466-480 peptides, and T cell responses were determined by an IFN-γ ELISPOT assay. Results correspond to data after subtraction of values obtained in control wells without peptide. **(E)** Purified splenic CD4 or CD8 T cells from BALB/c or C57BL/6J mice immunized with Omicron 446-488cc peptide vaccine plus exogenous T helper epitopes FIS or PADRE were co-cultured with irradiated splenocytes from naive mice and Omicron-derived peptides or exogenous T helper peptides FIS or PADRE. T cell responses were determined by an IFN-γ ELISPOT assay. Bars show mean +/- SEM (ns, not significant; *, P<0.05; **, P<0.01; ***, P<0.001). W, Wuhan; O, Omicron.

To overcome the poor immunogenicity of the Omicron-based 446-488cc peptide vaccine in mice, we repeated immunization experiments co-administering Omicron 446-488cc peptide with FIS and PADRE peptides, which are exogenous T helper epitopes for BALB/c and C57BL/6J mice, respectively (10), emulsified in Freund’s adjuvant. Under these conditions, potent binding IgG antibody responses were induced in both murine strains, recognizing 446-488cc peptides derived from the Wuhan and Omicron variants (**Figure 4A**). Moreover, these IgG antibodies recognized both Wuhan and Omicron RBD proteins, although Omicron RBD was significantly better recognized than the Wuhan RBD in both murine strains (**Figure 4B**). Importantly, these antibodies showed clear neutralizing activity against live SARS-CoV-2 Omicron, with mean 50% neutralizing titers of 5446 and 5852 in BALB/c and C57BL/6J mice, respectively (**Figure 4C**). Interestingly, T cell responses against endogenous T cell epitopes were somehow recovered, mainly in BALB/c mice, with a significant higher specificity for Omicron-based sequences (**Figure 4D**). These T cell responses against Omicron antigens were mediated by CD4 and CD8 T cells in the case of BALB/c mice, mainly directed against 446-460 epitope. As expected, responses against exogenous peptides FIS and PADRE were mediated by CD4 T cells, although in the case of peptide FIS poorer IFN-γ responses were observed (**Figure 4E**), in agreement with our previous studies (17).

## 4 Discussion

Breakthrough infections caused by the SARS-CoV-2 Omicron variant of concern occurring in vaccinated individuals allow viral transmission. Therefore, development of vaccines with a high capacity to induce responses recognizing this viral variant is necessary. We previously reported the immunogenicity and protective ability of 446-488cc, a vaccine based on a cyclized version of Wuhan peptide 446-488 (10). Here, to identify the best immunogen against Omicron variant, we have tested in immunized mice the cross-recognition capacity of antibodies induced by the Wuhan peptide 446-488cc, as well as the immunogenicity of an Omicron version of this peptide.

Regarding experiments testing recognition of variant antigens by antibodies induced using the original Wuhan sequence, two main issues arise: First, antibodies induced by Wuhan 446-488cc peptide recognize similarly antigens from Wuhan and Omicron variants (either peptide 446-488cc or RBD protein) in three different murine strains (BALB/c, C57BL/6J and transgenic K18-hACE2 mice). By contrast, Wuhan RBD-induced antibodies have a higher specificity for the sequence of the immunizing protein, with very low levels of antibodies against Omicron. Indeed, a decrease in the neutralization capacity against the different Omicron subvariants by antibodies induced by approved vaccines has been reported, which varies according to the type of vaccine administered (mRNA, adenoviral, protein or inactivated), and the subvariant considered (18–20). In our study, peptide and RBD vaccines induce polyepitopic responses, but the range of epitopes covered by the recombinant RBD vaccine is presumably wider, due to its higher molecular size. In this case, antibodies induced by a vaccine based on a larger antigen may be directed to other epitopes, without being so focused on the 446-488 region. Omicron variant contains more than 20 point mutations and deletions in S1 protein (11), with 15 of them located in the 319-541 sequence contained in the RBD vaccine. Our results indicate that, despite the presence of five mutations in the 446-488 region, these changes may be more conserved from an immune perspective, allowing a higher cross-recognition by peptide-induced antibodies. Conversely, mutated RBD epitopes located outside the 446-488 region may be less conserved, and antibodies against these epitopes induced after RBD vaccination would lose their capacity to cross-react with the Omicron epitopes. This higher number of mutations may also explain why recognition of less mutated RBD variants is not affected.

A second interesting issue relates to the capacity of our peptide vaccine to induce responses against epitopes not targeted when using the larger RBD vaccine. In C57BL/6J and K18-hACE2 mouse strains, the Wuhan 446-488cc peptide induced antibodies recognizing this region either in the context of the peptide or the RBD antigens. By contrast, when using RBD protein as immunogen, no antibodies are induced against 446-488 region. Vaccinated individuals and infected patients have been primed by large antigens. In an outbred heterogeneous population, it may not be uncommon the case of individuals lacking antibodies against the 446-488 region, as occurred in two of our murine strains. Indeed, analysis of sera from infected individuals showed variable and poor recognition of 446-488 peptide (10). However, by immunizing with the Wuhan 446-488cc peptide we induced antibodies in the three murine strains analyzed. These results indicate the capacity of this peptide vaccine to broaden the antibody repertoire towards cryptic epitopes, in this case, located at an immunologically conserved region, as opposed to larger antigen-based vaccines that presumably focus their repertoire against other more variable regions.

In another set of experiments, despite the prominent cross-recognition capacity of antibodies induced by the Wuhan 446-488cc peptide, we tested the suitability of a new vaccine based on the Omicron 446-488cc peptide. Surprisingly, poor antibody responses were induced in the murine strains tested (BALB/c and C57BL/6J), which we attribute to the lack of T helper epitopes. The original Wuhan 446-488cc peptide vaccine sequence contained T CD4 and CD8 epitopes (10) that are probably responsible for its high immunogenicity. Mutations occurring within these epitopes in the Omicron variant preclude activation of T cells in immunized mice, as predicted by MHC binding algorithms and confirmed by *in vitro* T cell assays. Thus, immunization in the presence of exogenous T helper epitopes rescued vaccine immunogenicity to levels achieved by the Wuhan 446-488cc peptide, which contains endogenous T cell epitopes. The immunization protocol carried out with the Omicron 446-488cc peptide, in addition to exogenous T cell epitopes, included a change in adjuvant (Freund’s adjuvant vs CpG/Alum) and an extra vaccine administration. Although these changes may potentially affect the results, we believe that addition of exogenous T helper epitopes is the main reason for the enhanced immunogenicity, since equivalent results were obtained with our previous Wuhan 446-488cc peptide vaccine when using any of these adjuvants and protocols (10). Antibody responses induced by the Omicron-based peptide vaccine, although cross-reacted against both variants, were more specific for Omicron, as demonstrated when using RBD as ELISA antigen. Moreover, this enhanced Omicron RBD recognition translated into robust Omicron SARS-CoV-2 neutralization titers, in the order of other previously reported by Omicron-directed vaccines in preclinical models (21–23) and above those titers reported in triple-vaccinated individuals with subsequent Omicron breakthrough infection (24). Regarding T cell responses, it is interesting to note the important conservation degree observed in predicted T cell epitopes in the human setting. This suggests that vaccines based on the Omicron 446-488cc sequence would retain immunogenicity, avoiding the use of exogenous T helper epitopes. Indeed, it has been described that T cell reactivity against Omicron is preserved in most vaccinated and infected individuals (4, 5), mainly for CD4 T cells (25), suggesting that the necessary T cell help for antibody induction would not be affected by sequence differences.

Finally, notwithstanding the high conservation level of the 446-488 sequence, the low levels of antibodies found in infected individuals suggest that it would be a region with a lower immune pressure, indicating that it could be a suitable target antigen for future subunit vaccines inducing humoral responses with a wider variant coverage.

## Data availability statement

The original contributions presented in the study are included in the article/supplementary materials. Further inquiries can be directed to the corresponding authors.

## Ethics statement

Animal studies were carried out after study approval by our institutional review committee (protocol 025-20). Animal procedures conformed with international guidelines and with Spanish law under the Royal Decree (RD 53/2013).

## Author contributions

BA, JL and PS conceived and designed experiments. BA, MR, NC, LS, JE, PP, GA and JG-A performed the *in silico*, *in vitro* and *in vivo* studies. BA, PP, GA, JG-A, ME, JL and PS analyzed data and contributed to their interpretation. JL and PS supervised the project. ME, JG-A, JL and PS received funding. All authors reviewed the manuscript, approved the final version to be published and agreed to be accountable for all aspects of the work in ensuring that questions related to the accuracy or integrity of any part of the work are appropriately investigated and resolved.
