# Erratum: Enhanced cross-recognition of SARS-CoV-2 Omicron variant by peptide vaccine-induced antibodies

**DOI:** 10.3389/fimmu.2023.1172427

**Published:** 2023-03-03

**Authors:** Belén Aparicio, Marta Ruiz, Noelia Casares, Leyre Silva, Josune Egea, Patricia Pérez, Guillermo Albericio, Mariano Esteban, Juan García-Arriaza, Juan J. Lasarte, Pablo Sarobe

**Affiliations:** ^1^Centro de Investigación Médica Aplicada (CIMA), Universidad de Navarra, Pamplona, Spain; ^2^Centro de Investigación Biomédica en Red de Enfermedades Hepáticas y Digestivas (CIBEREHD), Pamplona, Spain; ^3^Instituto de Investigaciones Sanitarias de Navarra (IdiSNA), Pamplona, Spain; ^4^Centro Nacional de Biotecnología (CNB), Consejo Superior de Investigaciones Científicas (CSIC), Madrid, Spain; ^5^Centro de Investigación Biomédica en Red de Enfermedades Infecciosas (CIBERINFEC), Madrid, Spain

**Keywords:** SARS-CoV-2, peptide vaccine, Omicron variant, cross-recognizing antibodies, conserved regions

Due to a production error, the **Author Contributions** statement was not updated to accurately reflect the contributions of the newly added authors PP, GA, JG-A and ME. The correct **Author Contributions** statement appears below.

## Author contributions

“BA, JL and PS conceived and designed experiments. BA, MR, NC, LS, JE, PP, GA and JG-A performed the *in silico*, *in vitro* and *in vivo* studies. BA, PP, GA, JG-A, ME, JL and PS analyzed data and contributed to their interpretation. JL and PS supervised the project. ME, JG-A, JL and PS received funding. All authors reviewed the manuscript, approved the final version to be published and agreed to be accountable for all aspects of the work in ensuring that questions related to the accuracy or integrity of any part of the work are appropriately investigated and resolved.”

The publisher apologizes for this mistake. The original article has been updated.

